# Evaluating home-delivered meal service programs for obesity: a randomized controlled approach

**DOI:** 10.29219/fnr.v70.12024

**Published:** 2026-05-07

**Authors:** Merve Çapaş, Aylin Ayaz

**Affiliations:** 1 Department of Community Nutrition, Faculty of Health Sciences, Erciyes University, Kayseri, Türkiye; 2 Department of Nutritional Sciences, Faculty of Health Sciences, Hacettepe University, Ankara, Türkiye

**Keywords:** home-delivered diet meal, dietary compliance, weight management, obesity, eating behavior, dietary counseling

## Abstract

**Background:**

Though a healthy diet is the most effective solution for obesity, it is hindered by two main barriers: difficulty in accessing or preparing healthy meals and lack of portion control knowledge.

**Objective:**

This study compared two different medical nutrition therapies (home-delivered diet meal service [HDMS] combined with dietary counseling and dietary counseling solely) in terms of anthropometric measurements, biochemical parameters and eating behaviors in women with overweight or obesity who participated in a weight loss program.

**Design:**

A non-randomized, controlled, parallel-group intervention study was conducted with 60 women aged 25–45 years, having a Body Mass Index (BMI) of 25–35 kg/m^2^, representing individuals with overweight and class I obesity. Participants were divided into two groups: an intervention group receiving HDMS combined with dietary counseling (*n* = 30) and a control group receiving dietary counseling solely (*n* = 30). Anthropometric measurements, bioelectrical impedance analysis (BIA), and dietary and physical activity records were monitored throughout the 8-week intervention, while comprehensive assessments including biochemical parameters (fasting blood glucose, homeostatic model assessment for insulin resistance (HOMA-IR), lipid profile), resting metabolic rate (RMR) (FITMATE), and eating behaviors assessed using the Three-Factor Eating Questionnaire (TFEQ) were conducted at baseline, week 4, and week 8.

**Results:**

Both groups showed significant reductions over time in body weight, BMI, waist circumference, hip circumference, body fat percentage, RMR, blood glucose, lipid parameters, and blood pressure values (*P* < 0.001). Although greater reductions were observed in the HDMS group, the differences between groups were not statistically significant (*P* > 0.05). When considering the group-time interaction, changes in waist circumference (*P* < 0.001), hip circumference (*P* < 0.05), RMR (*P* < 0.05), fasting blood glucose (*P* < 0.05), low-density lipoprotein (LDL), cholesterol (*P* < 0.05), and systolic blood pressure (*P* < 0.05) were found to be significant in the HDMS group. In addition, when examining eating behaviors, statistically significant changes were observed in the intervention group for both time effects and behavior outcomes. Uncontrolled eating and emotional eating behaviors decreased, while cognitive restraint increased (*P* < 0.001).

**Conclusion:**

Both interventions improved anthropometric and metabolic parameters, with greater reductions observed in the HDMS group; however, the differences between groups were not statistically significant. HDMS may be considered a practical approach to support weight management.

## Popular scientific summary

- A healthy diet is the most effective solution for obesity, but barriers such as difficulty accessing healthy meals and lack of portion control knowledge hinder success.
- The study shows that an 8 week Home Delivered Diet Meal Service (HDMS) is associated with improvements in weight, Body Mass Index (BMI), waist/hip circumference, body fat percentage, resting metabolic rate, and emotional eating.
- HDMS also supports glycemic control, lipid profile, and blood pressure, offering a practical alternative for those struggling with meal preparation and portion control, enhancing dietary adherence.

## Key Points

This study evaluated the effects of two dieting methods for managing obesity. While both approaches contributed to weight loss, the home-delivered meal service was found to be a better option compared to dietary counseling, as it facilitated dietary adherence more effectively.

Obesity, defined as abnormal or excessive fat accumulation, affected one in eight individuals globally in 2022. During this period, approximately 2.5 billion adults were classified as overweight, of whom 890 million were living with obesity, representing 43 and 16% of the global adult population, respectively. These figures highlight a more than twofold increase in adult obesity and a fourfold rise in adolescent obesity since 1990, underscoring the escalating prevalence of this global health concern (1). According to the Turkish Nutrition and Health Survey (TNHS 2017), 36.9% of adults (2) were overweight, 28.4% were individuals with obesity, and 3.8% were individuals with morbid obesity (3). The Turkish Population and Health Survey (TNSA) reports that only 26.6% of women have a normal body mass index (BMI) (4), with the obesity rate among women rising from 18.8% in 1998 to 30.3% in 2018 (5). Obesity, influenced by factors (6) such as age, gender, childbirth, smoking cessation, diet, and genetics, is linked to various health issues, including cardiovascular diseases, metabolic disorders, reproductive problems, and certain cancers (7–12). Losing 10% of initial body weight in individuals with obesity significantly reduces risk factors for chronic diseases, morbidity, and mortality (13, 14).

A combined approach of a low-energy diet increased physical activity, and behavioral therapy is effective for weight loss and maintenance (15). However, sustainability of behavioral changes in weight control is often hindered by limited access to healthy food and a lack of time or knowledge to prepare diverse, satisfying meals (16).

Recent studies highlight the need for innovative interventions that promote weight loss while addressing eating behaviors and dietary adherence (7, 17, 18). Home-delivered diet meal service (HDMS) offers pre-portioned, nutritionally balanced meals, addressing challenges in traditional weight loss programs related to meal preparation and portion control. However, the number of studies exploring the effects of HDMS on obesity and overall health parameters remains limited in the literatüre (16, 19–21).

HDMS has increasingly gained attention as a practical strategy to improve dietary adherence and support weight management in both clinical and real-world settings. Previous studies have demonstrated that providing nutritionally balanced, portion-controlled meals, often combined with dietary counseling, can lead to improvements in dietary intake, metabolic parameters, and cardiovascular risk factors (16, 22, 23). In particular, comparative studies evaluating home-delivered diet and dietary counseling have reported that both approaches improve cardiometabolic outcomes; however, greater reductions may be observed in individuals receiving home-delivered diet services, potentially due to improved adherence (24). In addition, interventions involving delivered meals have been associated with improved glycemic control and dietary compliance in individuals with chronic conditions (21, 25). Moreover, structured meal delivery systems may reduce common barriers such as meal planning and preparation burden, which are frequently reported as key obstacles to sustained dietary change (19, 20). Women were specifically selected for this study due to sex-specific differences in body composition, eating behaviors, and weight loss responses, which may influence the effectiveness of dietary interventions. Therefore, this study aimed to compare two different medical nutrition therapies (HDMS and a control group receiving dietary counseling solely) in terms of anthropometric measurements, biochemical parameters, and eating behaviors, as well as to evaluate their effects on the Three-Factor Eating Questionnaire (TFEQ) and the associations between changes in eating behaviors.

## Methods

### Study design and setting

This study was designed as a non-randomized, controlled, parallel-group intervention study conducted at a private health and nutrition center in Kayseri, Türkiye. The study compared the effects of dietary counseling solely versus combined dietary counseling and HDMS in women with overweight and obesity seeking weight loss treatment. The recruitment of study participants began in March 2018 and was completed in December 2018. The study was performed in compliance with the World Medical Association (WMA) Declaration of Helsinki: Ethical Principles for Medical Research Involving Human Subjects. Ethical approval was obtained from the XXX University Clinical Research Ethics Committee on February 9, 2018 (Decision No: 2018/64). All participants provided informed consent prior to their involvement in the study.

### Study population

Female individuals aged 25–45 years with a BMI of 25–35 kg/m^2^, who sought weight loss treatment at the study center, were recruited for the study. Inclusion criteria included being aged 25–45 years, having a BMI between 25 and 35 kg/m^2^, and having a sedentary to moderate physical activity level. Participants within this BMI range were selected to represent individuals with overweight and class I obesity, a group commonly targeted in lifestyle-based weight management interventions. Exclusion criteria included pregnancy, breastfeeding, chronic diseases, alcohol consumption, common food allergies, recent weight loss exceeding 5% within the past 3 months, use of weight-loss medications, steroid therapy, and a history of acute or chronic inflammatory diseases. The sample size was calculated using G*Power software (version 3.1) and Minitab, based on expected differences in body weight considering results from previous studies (16, 22), with a significance level of 0.05 and a power of 0.80, resulting in a required sample size of 60 participants. Consistent results were obtained from both methods, and the final sample size determined by G*Power was adopted for the study.

### Group allocation

**Fig. 1:**
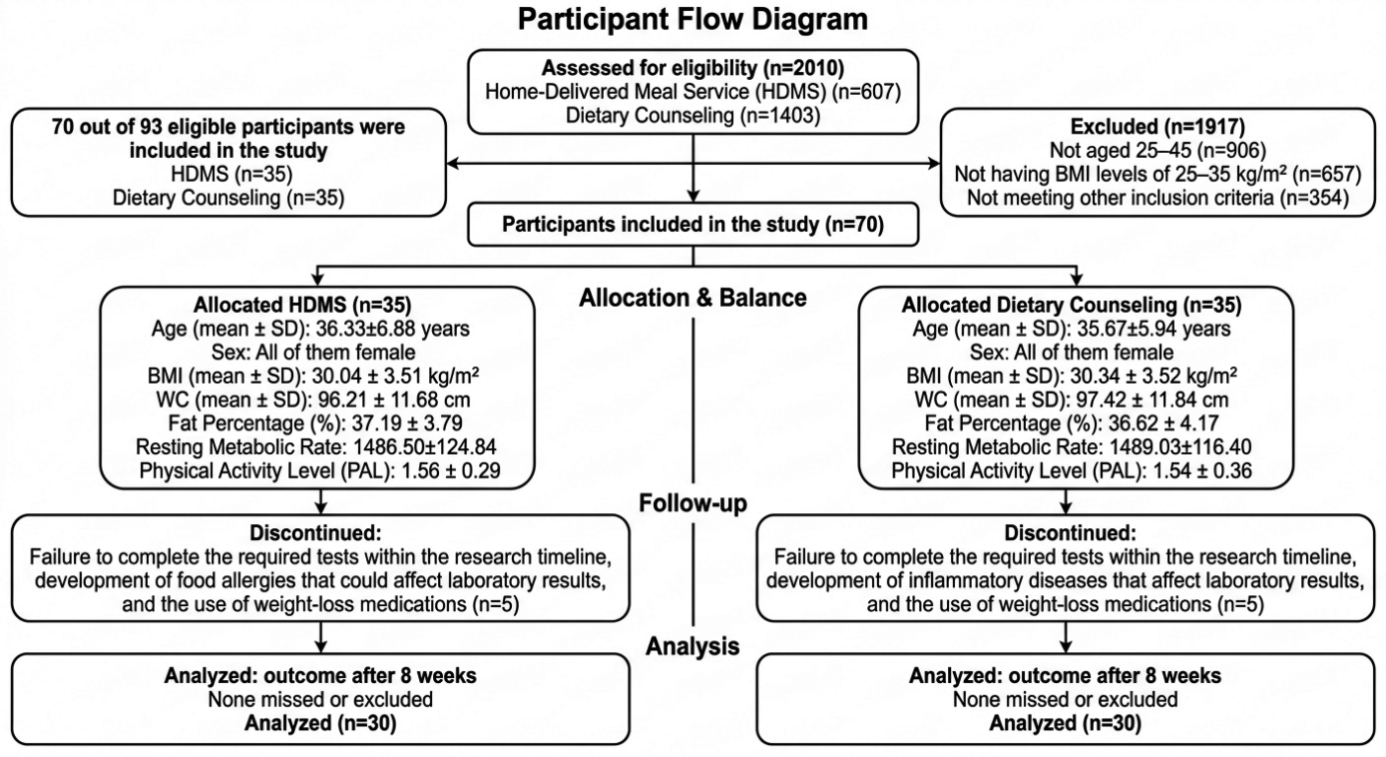
Study flow diagram showing participants’ recruitment, allocation, and follow-up.

Participants who met the inclusion criteria were informed about both HDMS and dietary counseling options. Individuals were allowed to choose their preferred intervention based on personal preference. Following this selection, participants were allocated to either the intervention group receiving HDMS combined with dietary counseling or to the control group receiving dietary counseling solely. Due to differences in group sizes, a balanced number of participants from each group were included in the final sample to ensure comparability between groups. A total of 60 participants completed the study (30 in the intervention group and 30 in the control group). Ten participants were excluded during follow-up due to failure to complete required tests, development of acute inflammatory diseases or food allergies, and the use of weight-loss medications. The participant flow diagram is presented in Fig. 1.

### Blinding

Due to the nature of the intervention, blinding of participants and the dietitian was not feasible. Therefore, the study was conducted as an open-label trial. However, to minimize potential bias, the statistician responsible for data analysis was blinded to group allocation throughout the analysis process.

### Detailed protocol

**Fig. 2:**
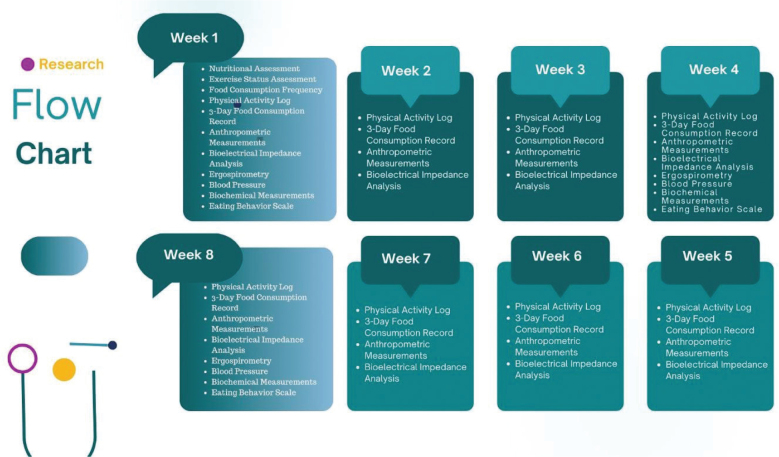
Research flow chart.

This study was conducted using a parallel-group design with an allocation ratio of 1:1. The intervention group received HDMS in addition to dietary counseling, while the control group received dietary counseling solely. Using face-to-face interview techniques, the researcher administered a questionnaire covering socio-demographic characteristics, frequency of food consumption, 3-day food consumption records (including one weekend day), physical activity records and the TFEQ. Anthropometric measurements (height, body weight, waist, neck, and hip circumferences) were obtained, and body composition was assessed using bioelectrical impedance analysis (BIA). Resting metabolic rate (RMR) was measured using an ergospirometer. Waist-to-hip ratio and BMI values were calculated. Dietary energy and macronutrient intake were assessed using 3-day food consumption records. The collected dietary data were analyzed using BEBİS (Nutrition Information System) software to calculate daily energy and macronutrient intake. Physical activity levels were assessed using 3-day physical activity records collected alongside dietary intake data. Participants reported their daily activities during these days, and physical activity levels were classified as sedentary to moderate according to Physical Activity Level (PAL) and Physical Activity Ratio (PAR) calculations based on reported activities (26). The research flow chart is presented in Fig. 2.

### Dietary interventions

**Fig. 3:**
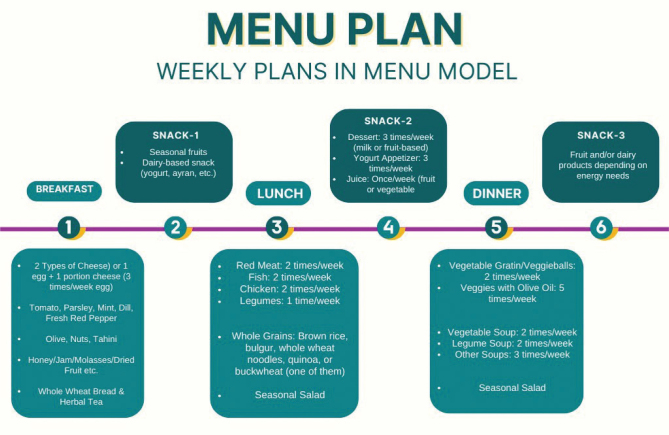
Dietary intervention protocol.

A balanced nutrition plan (comprising 55–60% carbohydrates, 15–20% protein, and 25–30% fat) was developed based on participants’ RMR, taking into account age, gender, physical activity, and overall health status. To achieve a target weight loss of 0.5–1 kg per week, daily energy intake was reduced by 500–1,000 kcal, in accordance with clinical guidelines (27). Both study groups followed a dietary regimen consisting of three main meals and three snacks per day, including fish twice per week, legumes once per week, poultry twice per week, and red meat twice per week. Eggs were included in breakfast three times per week. The dietary intervention protocol is presented in Fig. 3. Meals for the intervention group were prepared and delivered daily by trained culinary staff. Participants in the control group prepared their own meals based on a prescribed dietary plan designed to ensure consistency in meal structure and content. Both groups received training in food preparation and portion control. Participants were also instructed to consume at least 2 L of calorie-free fluids daily and to maintain stable physical activity level throughout the study.

### Outcome variables

#### Antropometric measurements

Participants’ body weight, height, waist, neck and hip circumferences were measured to calculate waist-to-hip ratios and BMI, with body composition analyzed weekly over 8 weeks. Body composition was assessed using a TANITA MC 780 BIA device. Measurements were taken under standardized conditions, with participants lightly clothed, barefoot, and following fasting requirements (28). Height was measured to a 1 mm precision using a SECA stadiometer, with participants standing upright and aligned in the Frankfurt plane (29, 30). BMI was calculated as weight (kg) divided by height squared (m^2^) and classified per WHO guidelines (31). Waist circumference, used to assess chronic disease risk, was measured three times between the lowest rib and iliac crest with participants standing upright and abdomen relaxed (30). Hip circumference was taken from the right side at the highest point of the hip, and also repeated three times for accuracy (28). Neck circumference was measured at the lower border of the larynx with participants standing in the Frankfurt plane (32, 33). BIA was performed using the TANITA MC 780 device, which measures lean body mass and body fat percentage with 0.1 kg accuracy, suitable for individuals aged 5–99 years, and with a 270 kg capacity. Participants avoided heavy activity for 24–48 h, alcohol for 24 h, food for at least 3 h, and fluids such as tea/coffee for 4 h before testing (34, 35). RMR was assessed using the FITMATE device via indirect calorimetry, with measurements taken after a 10–12-h, in a supine position, in a room maintained at 22–26°C to ensure accuracy (36, 37).

### Biochemical analysis

During the initial interview, participants provided recent biochemical test results, including fasting blood glucose, lipid profile, liver enzymes (AST, ALT), uric acid, creatinine, complete blood count, TSH, FT4, ferritin, and B12 levels from Kayseri Central Laboratory. Systolic and diastolic blood pressure were also recorded. As serum insulin and homeostatic model assessment for insulin resistance (HOMA-IR), tests were not free of charge at this laboratory, 5 mL of blood was drawn, stored at –20°C, and analyzed at a private lab in Ankara, with costs covered by the researcher. Participants repeated the same tests at weeks 4 and 8. Serum insulin was measured via ECLIA using the Roche E170 device, with normal levels being 2–20 µIU/mL, and HOMA-IR calculated using the formula: HOMA = (Fasting Insulin × Fasting Glucose)/405, with values below 2.7 considered normal (38).

### Three-factor eating questionnaire

The ‘Three-Factor Eating Questionnaire’ (TFEQ), developed by Karlsson et al. (39), assesses three eating behaviors: Cognitive Restraint, Uncontrolled Eating, and Emotional Eating (40). It includes 18 items, validated in Türkiye by Kiraç et al. (6). Items 1–13 are scored from 4 to 1, items 14 and 17 from 1 to 4, and item 18 has a mixed scoring pattern. Scores for ‘Uncontrolled Eating’ (items 1, 4, 5, 7, 8, 9, 13, 14, 17) range from 9–36, ‘Cognitive Restraint’ (items 2, 11, 12, 15, 16, 18) from 6–24, and ‘Emotional Hunger’ (items 3, 6, 10) from 3 to 12 (41).

### Compliance

During the 8-week intervention, participants attended weekly (30 min) and monthly (60 min) sessions. Weekly evaluations included anthropometric measurements, BIA, physical activity, and dietary intake. Biochemical parameters, blood pressure, metabolic rate, and dietary habits were assessed at baseline, week 4, and week 8.

### Statistical analysis

Data obtained from the study were analyzed using IBM SPSS Statistics 25.0 (IBM Corp. Released 2017). The normality of numerical variables was assessed using the Shapiro–Wilk test and Q-Q plots. Within-group changes over time (time effect) and group × time interactions were analyzed using repeated measures two-way analysis of variance (ANOVA), for normally distributed variables, followed by Bonferroni post hoc tests. Inter-group comparisons were performed using the independent two-sample *t*-test for normally distributed variables and the Mann–Whitney U test for non-normally distributed variables. A *P*-value of < 0.05 was considered statistically significant (42).

## Results

### Participants

This study was conducted on women with overweight and obesity aged 25–45, with an intervention group receiving HDMS and a control group receiving only dietary counseling. The mean age of the intervention group was 36.33 ± 6.88 years, while that of the control group was 35.67 ± 5.94 years (*P* > 0.05). Also, the mean BMI of the intervention group was 30.04 ± 3.51 kg/m^2^, while that of the control group was 30.34 ± 3.52 kg/m^2^ (*P* > 0.05). No statistically significant differences were observed between the groups in terms of age, educational background, occupation, marital status, or economic condition (*P* > 0.05). The majority of participants were married, had a favorable economic status, and all resided in urban areas (Table 1). The statistical analysis revealed that the data were homogeneously distributed within the groups, supporting the validity of comparisons made throughout the study.

**Table 1:** General descriptive characteristics of individuals

General characteristics	Control (*n* = 30)		Intervention (*n* = 30)		*P*
	*n*	%	*n*	%	
**Age, years** (mean ± SD)	35.67 ± 5.94		36.33 ± 6.88		0.761
**Education level**					
Primary school	-	-	1	3.3	
Secondary school	4	13.3	-	-	
High school	9	30.0	10	33.3	0.295^†^
University	15	50.0	17	56.7	
Post Graduate	2	6.7	2	6.7	
**Profession**					
Officer	5	16.7	3	10.0	
Self-employment	4	13.3	2	6.7	
Housewife	15	50.0	18	60.0	0.205^†^
Unemployed	3	10.0	-	-	
Other (student, private sector)	3	10.0	7	23.3	
**Marital status**					
Married	23	76.7	23	76.7	
Single	7	23.3	7	23.3	1.000^†^
**Economic situation**					
Income equals to expenses	2	6.7	5	16.7	
Income more than expenses	28	93.3	25	83.3	0.424^†^
**Place of residence**					
City	30	100.0	30	100.0	-
**Obesity problem in the family**					
No	19	63.3	17	56.7	
Yes	11	36.7	13	43.3	0.792^+^
**Having previously used diet therapy to lose weight**					
No	2	6.7	3	10.0	
Yes	28	93.3	27	90.0	0.706^‡^
**Change in body weight in the last 6 months**					
Unchanged	9	30.0	14	46.7	
Increased	14	46.7	13	43.3	0.260^†^
Decreased	7	23.3	3	10.0	

Column percentages calculated.

† Pearson Chi-Square test ^+^Chi-Square test with continuity correction

‡ Fisher’s exact test.

### Eating habits, physical activity patterns, and related conditions

When comparing dietary habits, 46.7% of the intervention group and 20.0% of the control group had regular meal times on weekdays; on weekends, this was 26.7% versus 13.3% (*P* > 0.05). Eating out was common, with 89.7% of the intervention group and 76.7% of the control group doing so, mostly at canteens or restaurants for breakfast (*P* > 0.05). Lunch was more often eaten at restaurants by the intervention group (66.7%) compared to the control group (50.0%), with a significant difference in kebab-grilled versus fast food choices (*P* = 0.007). Dinner preferences differed, but not significantly (*P* = 0.060). Meal skipping was higher for lunch in the intervention group (61.9%) and breakfast in the control group (52.0%), with a significant difference in meal-skipping frequency (*P* = 0.043). Snack skipping was more frequent in the intervention group (76.7%) than in the control group (56.7%), with different snack preferences but no significant difference was found (*P* > 0.05). There were no significant differences between the groups regarding previous dieting, weight changes, or adherence to diet recommendations (*P* > 0.05). Regular exercise was low overall (78.3%), with no significant difference between groups (*P* > 0.05). The groups were distributed homogeneously, with no potential bias affecting the intervention.

### Anthropometric measurements and body composition

**Fig. 4:**
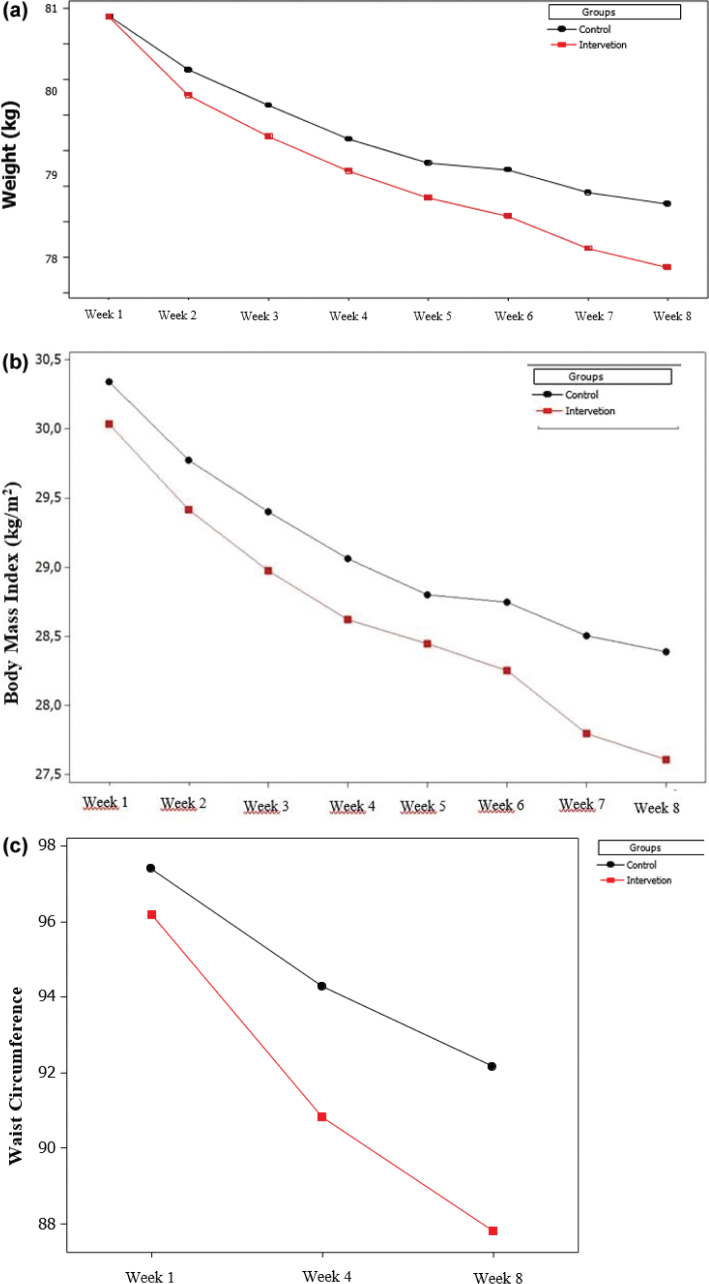
Changes in anthropometric measurements by week for intervention and control groups: (a) weight (b) body mass index (BMI) (c) waist circumference.

The study results indicated significant changes over time in both groups regarding anthropometric measures (body weight, BMI, waist circumference) and body composition (body fat percentage, lean mass, body fluid mass). Both groups experienced weight loss over 8 weeks, but the group × time interaction effect for weight and BMI was not statistically significant (*P* > 0.05), indicating similar reductions in both groups. However, significant group × time interaction effects were observed for waist circumference (*P* < 0.001) and hip circumference (*P* = 0.002), suggesting differing changes between groups, with the intervention group showing distinct outcomes. Body fat percentage and body fluid mass showed significant reductions over time in both groups (*P* < 0.001), but the group × time interaction effect was not significant (*P* > 0.05). Neck circumference demonstrated a significant interaction effect (*P* < 0.05), while the waist-to-hip ratio did not (*P* = 0.493). For RMR, the group × time interaction effect was significant (*P* < 0.05), reflecting different changes between the groups over time, although baseline and 8-week RMR values were similar (*P* = 0.501). Anthropometric measurements and body composition data are presented in Table 2, and the changes observed during 8 weeks are also graphically shared in Fig. 4.

**Table 2:** Pre-diet and post-diet anthropometric measurement according to dieting method

Variables	Control (*n* = 30) $\bar{x}$ ± *ss*	Intervention (*n* = 30) $\bar{x}$ ± *ss*	Between groups *P*-value
**Body weight (kg)**			
Week 1	80.79 ± 9.71^a^	80.76 ± 9.81^a^	*P* = 0.993
Week 8	75.49 ± 9.01^b^	73.71 ± 9.73^b^	*P* = 0.464
***Within Groups (Week 1 to Week 8)*** ^*^	***P* < 0.001**	***P* < 0.001**	
***Body Mass Index (kg/m***^2^***)***			
Week 1	30.34 ± 3.52^a^	30.04 ± 3.51^a^	*P* = 0.737
Week 8	28.39 ± 3.47^b^	27.61 ± 3.33^b^	*P* = 0.376
***Within Groups (Week 1 to Week 8)*** ^*^	***P < 0.001***	***P < 0.001***	
**Waist Circumference (cm)**			
Week 1	97.42 ± 11.84^a^	96.21 ± 11.68^a^	*P = 0.692*
Week 8	92.17 ± 11.83^b^	87.82 ± 12.03^b^	*P = 0.163*
***Within Groups (Week 1 to Week 8)*** ^*^	***P* < 0.001**	***P* < 0.001**	
***Hip Circumference (cm)***			
Week 1	115.35 ± 7.46^a^	114.43 ± 9.63^a^	*P = 0.682*
Week 8	109.18 ± 7.43^b^	105.40 ± 9.75^b^	*P = 0.096*
***Within Groups (Week 1 to Week 8)*** ^*^	***P* < 0.001**	***P* < 0.001**	
***Neck Circumference (cm)***			
Week 1	36.82 ± 2.60^a^	36.57 ± 2.54^a^	*P = 0.707*
Week 8	34.80 ± 2.22^b^	34.73 ± 2.02^b^	*P = 0.903*
***Within Groups (Week 1 to Week 8)*** ^*^	***P* < 0.001**	***P* < 0.001**	
***Body Fat Percentage (%)***			
Week 1	36.62 ± 4.17^a^	37.19 ± 3.79^a^	*P* = 0.584
Week 8	34.04 ± 4.88^b^	33.34 ± 4.80^b^	*P* = 0.578
***Within Groups (Week 1 to Week 8)*** ^*^	***P < 0.001***	***P < 0.001***	
***Resting Metabolic Rate (kcal)***			
Week 1	1489.03 ± 116.40^a^	1486.50 ± 124.84^a^	*P* = 0.935
Week 8	1436.10 ± 106.71^b^	1415.96 ± 123.22^c^	*P* = 0.501
***Within Groups (Week 1 to Week 8)*** ^*^	***P* < 0.001**	***P* < 0.001**	

* Repeated measurements were performed with two-way analysis of variance (Repeated Measures Two way ANOVA). Values marked with different letters in the 1st and 8th weeks in the case and control groups were found to be statistically significant (*P* <0.05).

### Biochemical parameters and blood pressure

A significant group × time interaction was observed for fasting blood glucose (*P* = 0.015) and LDL cholesterol levels (*P* = 0.037), indicating that changes in these parameters over time differed between the intervention and control groups. However, for HOMA-IR, total cholesterol, HDL-C only a significant time effect was found (*P* < 0.001), suggesting that these parameters changed significantly over time within both groups without any significant group × time interaction. Furthermore, a significant group × time interaction was found for systolic blood pressure (*P* = 0.024). For diastolic blood pressure, there was a significant difference between groups at baseline (*P* = 0.042), and a significant time effect was observed within groups (*P* < 0.001), though the group × time interaction was not significant (*P* = 0.109). These findings indicate that while some parameters exhibited group-specific changes over time, others were primarily influenced by time, independent of group assignment. Biochemical parameters and blood pressure data are presented in Table 3.

**Table 3:** Pre-diet and post-diet biochemical parameters and blood pressure according to dieting method

Variables	Control (*n* = 30) $\bar{x}$ ± *ss*	Intervention (*n* = 30) $\bar{x}$ ± *ss*	Between Groups *P*-value
**Fasting Blood Glucose (mg/dL)**			
Week 1	105.33 ± 29.30^a^	109.48 ± 31.27^a^	*P* = 0.607
Week 8	102.73 ± 29.25^c^	105.89 ± 30.82^c^	*P* = 0.693
***Within Groups (Week 1 to Week 8)*** ^*^	***P* < 0.001**	***P* < 0.001**	
***HOMA-IR***			
Week 1	2.21 ± 0.65^a^	2.30 ± 0.70^ac^	*P* = 0.580
Week 8	2.01 ± 0.60^b^	2.16 ± 0.66^bd^	*P* = 0.360
***Within Groups (Week 1 to Week 8)*** ^*^	***P < 0.001***	***P* = 0.001**	
**Total Cholesterol (mg/dL)**			
Week 1	215.90 ± 38.34^a^	219.00 ± 47.45^a^	*P* = 0.785
Week 8	207.30 ± 37.24^b^	206.82 ± 44.03^b^	*P* = 0.964
***Within Groups (Week 1 to Week 8)*** ^*^	***P* < 0.001**	***P* < 0.001**	
***LDL-C (mg/dL)***			
Week 1	130.13 ± 33.26^a^	140.41 ± 38.98^a^	*P* = 0.280
Week 8	122.00 ± 31.81^b^	133.52 ± 37.21^b^	*P* = 0.206
***Within Groups (Week 1 to Week 8)*** ^*^	***P < 0.001***	***P < 0.001***	
***HDL-C (mg/dL)***			
Week 1	49.76 ± 10.36^a^	46.83 ± 10.35^a^	*P* = 0.286
Week 8	52.03 ± 9.93^b^	48.76 ± 10.17^b^	*P* = 0.220
***Within Groups (Week 1 to Week 8)*** ^*^	***P < 0.001***	***P < 0.001***	
***Systolic Blood Pressure (mmHg)***			
Week 1	122.47 ± 10.56^a^	128.04 ± 9.78^ad^	*P* = 0.052
Week 8	120.30 ± 8.87^b^	125.79 ± 9.17^c^	*P* = 0.030
***Within Groups (Week 1 to Week 8)*** ^*^	***P < 0.001***	***P < 0.001***	

* Repeated measurements were performed with two-way analysis of variance (Repeated Measures Two-way ANOVA). Values marked with different letters in the 1st and 8th weeks in the case and control groups were found to be statistically significant (*P* < 0.05).

### Dietary energy and macronutrient intake

Although energy intake increased in both groups, the increase was minimal in the intervention group, while it was nearly 200 kcal higher in the control group (*P* < 0.001), suggesting better diet adherence in the intervention group. Protein and fat intake increased, while carbohydrate intake decreased in both groups (group × time effect significant for all three variables, *P* < 0.001). Dietary energy and macronutrient intake are presented in Table 4.

**Table 4:** Pre-diet and post-diet dietary energy and macronutrient intake according to dieting method

Variables	Control (*n* = 30) $\bar{x}$ ± *ss*	Intervention (*n* = 30) $\bar{x}$ ± *ss*	Between Groups *P*-value
**Energy (kcal)**			
Week 1	1747.16 ± 172.09^a^	1436.54 ± 209.20^c^	***P* < 0.001**
Week 8	1902.86 ± 173.33^b^	1484.96 ± 193.75^d^	***P* < 0.001**
***Within Groups (Week 1 to Week 8)*** ^*^	***P* < 0.001**	***P* < 0.001**	
**Protein (%)**			
Week 1	17.69 ± 0.76^a^	19.20 ± 0.92^c^	***P* < 0.001**
Week 8	19.11 ± 0.46^b^	20.45 ± 0.51^d^	***P* < 0.001**
***Within Groups (Week 1 to Week 8)*** ^*^	***P < 0.001***	***P < 0.001***	
***Carbohydrate (%)***			
Week 1	49.40 ± 1.80^a^	54.01 ± 1.93^c^	***P* < 0.001**
Week 8	40.57 ± 1.93^b^	47.49 ± 1.79^d^	***P* < 0.001**
***Within Groups (Week 1 to Week 8)*** ^*^	***P < 0.001***	***P < 0.001***	
***Fiber (g)***			
Week 1	22.55 ± 3.33^a^	32.76 ± 5.14^c^	***P* < 0.001**
Week 8	28.78 ± 1.93^b^	28.29 ± 3.06^b^	*P* = 0.467
***Within Groups (Week 1 to Week 8)*** ^*^	***P < 0.001***	***P < 0.001***	
**Fat (%)**			
Week 1	32.89 ± 1.78^a^	26.55 ± 1.73^c^	***P* < 0.001**
Week 8	40.01 ± 2.05^b^	32.03 ± 1.94^d^	***P* < 0.001**
***Within Groups (Week 1 to Week 8)*** ^*^	***P* < 0.001**	***P* < 0.001**	
**Saturated fatty acid (g)**			
Week 1	23.75 ± 3.47^a^	16.37 ± 2.48^c^	***P* < 0.001**
Week 8	33.18 ± 5.78^b^	14.75 ± 4.03^d^	***P* < 0.001**
***Within Groups (Week 1 to Week 8)*** ^*^	***P < 0.001***	***P < 0.001***	
**Polyunsaturated Fatty Acids (g)**			
Week 1	15.64 ± 2.33^a^	8.74 ± 1.77^c^	***P* < 0.001**
Week 8	14.08 ± 1.46^b^	9.92 ± 1.29^d^	***P* < 0.001**
***Within Groups (Week 1 to Week 8)*** ^*^	***P < 0.001***	***P < 0.001***	

* Repeated measurements were performed with two-way analysis of variance (Repeated Measures Two-way ANOVA). Values marked with different letters in the 1st and 8th weeks in the case and control groups were found to be statistically significant (*P* < 0.05).

### Three-Factor Eating Questionnaire

At baseline, there were no significant differences between the intervention and control groups in uncontrolled eating, cognitive restraint, and emotional eating scores. By week-8, the intervention group showed a significant reduction in uncontrolled eating (*P* = 0.008) and emotional eating (*P* = 0.002), while cognitive restraint increased significantly compared to the control group (*P* = 0.001). The group × time effect was statistically significant for all three behaviors (uncontrolled eating: *P* = 0.003, cognitive restraint: *P* < 0.001, emotional eating: *P* < 0.001). Results from TFEQ are presented in Table 5.

**Table 5:** Pre-diet and post-diet eating behavior according to dieting method

Variables	Control (*n* = 30) $\bar{x}$ ± *ss*	Intervention (*n* = 30) $\bar{x}$ ± *ss*	Between Groups *P*-value
**Uncontrolled eating**			
Week 1	23.03 ± 4.88^a^	21.37 ± 6.87^a^	*P* = 0.283
Week 8	23.23 ± 4.90^a^	19.30 ± 6.04^b^	***P* = 0.008**
***Within Groups (Week 1 to Week 8)*** ^*^	*P* = 0.698	***P* < 0.001**	
**Cognitive restraint**			
Week 1	14.60 ± 3.79^a^	14.33 ± 3.69^a^	*P* = 0.783
Week 8	14.97 ± 3.98^a^	18.47 ± 3.53^b^	***P* = 0.001**
***Within Groups (Week 1 to Week 8)*** ^*^	*P* = 0.743	***P* < 0.001**	
**Emotional eating**			
Week 1	9.53 ± 2.18^a^	8.50 ± 3.10^a^	*P* = 0.141
Week 8	10.13 ± 2.36^b^	7.97 ± 2.68^c^	***P* = 0.002**
***Within Groups (Week 1 to Week 8)*** ^*^	***P* < 0.001**	***P* < 0.001**	

* Repeated measurements were performed with two-way analysis of variance (Repeated Measures Two-way ANOVA). Values marked with different letters in the 1st and 8th weeks in the case and control groups were found to be statistically significant (*P* < 0.05).

## Discussion

Obesity has increased dramatically over the past decades and remains a major contributor to chronic diseases such as heart disease and diabetes. Although adopting a healthy eating is considered the most effective strategy for weight management, practical barriers such as limited access to healthy meals and insufficient portion control knowledge often hinder its behavioral sustainability (43). In this context, HDMS have emerged as a practical alternative to support dietary adherence, particularly for individuals who experience difficulties in meal preparation and portion control (18). Previous studies indicate that HDMS interventions are commonly used to improve dietary habits and quality of life, enhance adherence to dietary programs, and promote favorable metabolic outcomes, particularly in individuals with cardiometabolic risk (16, 17, 19, 20, 22, 23, 25, 44–47):

This study examined the effects of HDMS combined with dietary counseling compared with dietary counseling alone on anthropometric, metabolic, and eating behavior outcomes. The findings indicate that both approaches were effective in improving health-related parameters; however, greater improvements were generally observed in the HDMS group, suggesting an added benefit of structured meal provision.

### Anthropometric measurements and body composition

The results of this study are in line with previous research demonstrating that structured dietary interventions can positively influence anthropometric outcomes (48, 49). Several studies have reported significant reductions in body weight, BMI, and waist circumference following HDMS interventions. For example, Gleason et al. (22) reported 3.7 kg weight loss and 0.9-unit decrease in BMI after an 8-week HDMS Program, while The Dietary Approaches to Stop Hypertension (DASH)-based intervention also showed greater improvements in BMI when supported by meal delivery systems (23). Similarly, De Ferranti et al. (50) observed reduction in waist circumference among adolescents. In addition, Hu et al. (51) reported that increased abdominal fat is strongly associated with higher cardiometabolic risk, while Choi et al. (52) highlighted that gynoid fat distribution is linked to a lower risk of type 2 diabetes. Mediterranean diet interventions supported by structured meal plans have also been shown to result in substantial reductions in abdominal adiposity, particularly among menopausal women (53). Consistent with these findings, this study demonstrated greater reductions in body weight, BMI, waist circumference, and body fat percentage in the HDMS group. These effects may be explained by the structured nature of HDMS, which reduces variability in dietary intake and facilitates portion control. By minimizing the need for meal planning and preparation, HDMS may also reduce cognitive and behavioral barriers to adherence, thereby enhancing the effectiveness of dietary interventions. In addition, the supportive role of structured meal provision may contribute to improved metabolic efficiency, which is reflected in favorable changes in RMR (54).

### Biochemical parameters and blood pressure

Obesity is closely associated with adverse cardiometabolic profiles, including dyslipidemia, impaired glucose metabolism, and elevated blood pressure (55–57). Previous studies have shown that HDMS interventions can contribute to improvements in these parameters. De Ferranti et al. (50) reported reductions in total cholesterol, HOMA-IR, and systolic blood pressure following HDMS interventions, while Gleason et al. (22) observed improvements in lipid profiles. Similarly, DASH-based meal delivery interventions have demonstrated effectiveness in reducing blood pressure (25), and Mediterranean dietary approaches have been associated with improvements in triglyceride levels and cardiovascular risk markers (53). Two kg weight loss was associated with reductions of 17.8 mg/dL in total cholesterol and 14.1 mg/dL in LDL (25, 47). Low-carbohydrate diets have also been shown to improve fasting glucose and triglyceride levels (44). In addition, randomized trials highlight the role of HDMS in reducing LDL and triglycerides, thereby supporting better glycemic control (16, 58). HDMS may also enhance adherence through portion control and improved dietary fat quality, contributing to reductions in systolic blood pressure (46). The findings of this study are consistent with these reports, indicating improvements in biochemical parameters and blood pressure in both groups, with more pronounced changes in the HDMS group. These improvements may be attributed to the consistent intake of nutritionally balanced meals, which support glycemic control and lipid regulation. Furthermore, structured dietary intake may reduce fluctuations in energy and nutrient consumption, thereby contributing to more stable metabolic responses. The combination of HDMS with dietary counseling likely reinforces these effects by providing both practical and educational support for behavioral change.

### Dietary energy and macronutrient intake

Energy intake and macronutrient distribution play a central role in weight management and dietary adherence (7). Previous research has shown that HDMS interventions are associated with reductions in daily energy intake and improvements in dietary composition. For instance, an 8-week HDMS intervention reported a reduction of approximately 255 kcal per day, accompanied by increased carbohydrate intake and decreased fat intake (22). Similarly, DASH-based interventions have demonstrated that structured meal provision can effectively reduce energy intake by improving portion control (23). In line with these findings, this study observed increases in energy intake in both groups over time; however, the increase was smaller in the HDMS group, suggesting relatively better adherence to the prescribed dietary plan. Dietary adherence in this study was not assessed using a specific adherence scale but was interpreted indirectly based on changes in energy intake, macronutrient distribution, and eating behavior scores. The smaller deviation from prescribed energy intake in the HDMS group may therefore reflect improved compliance facilitated by structured meal provision. Nevertheless, this interpretation should be considered with caution, as adherence was not directly measured. Variations in macronutrient intake observed in the literature further highlight the complexity of dietary adherence. For example, some studies have reported increased fat intake despite energy restriction (45) while others have demonstrated shifts in macronutrient distribution influenced by dietary patterns and individual preferences (59–63). These findings suggest that even within structured programs, individual variability and local dietary habits play a role in shaping dietary outcomes.

### Three-Factor Eating Questionnaire

Eating behaviors are important determinants of long-term weight management. Uncontrolled eating is defined as a tendency to overeat due to a loss of self-control (64–66), while emotional eating refers to consuming high-energy foods triggered by emotional states such as stress or joy, without physiological hunger (67). Moreover, cognitive restraint reflects conscious efforts to regulate food intake and has been associated with neural activity in food reward and inhibitory control regions (68, 69). Işgin et al. (70) reported lower cognitive restraint scores in lean individuals, while Konttinen et al. (71) demonstrated associations between emotional eating and BMI, as well as psychological factors. In this study, participants in the HDMS group exhibited reductions in uncontrolled and emotional eating behaviors, along with an increase in cognitive restraint. These findings suggest that structured meal plans may contribute to improved behavioral regulation, potentially by reducing the burden of food-related decision-making and limiting exposure to environmental triggers associated with overeating. In addition, more standardized meal timing and composition may support the development of more consistent eating patterns, which could facilitate healthier behavioral responses to food.

### Interpretation, strengths, and limitations

The findings of this study suggest that the beneficial effects observed in the intervention group may not be attributable to HDMS alone, but rather to the combined effect of structured meal provision and dietary counseling. While dietary counseling provides the knowledge and guidance necessary for behavior change, HDMS facilitates the practical implementation of these recommendations by reducing barriers related to meal preparation and portion control. This study has several strengths. It was conducted in a real-world clinical setting, enhancing the applicability of the findings. In addition, multiple outcome domains, including anthropometric, biochemical, and behavioral parameters, were assessed simultaneously, providing a comprehensive evaluation of intervention effects. The combination of HDMS and dietary counseling also reflects a pragmatic approach to obesity management. However, several limitations should be considered. The study was non-randomized, and participants selected their preferred intervention, which may introduce selection bias. Dietary adherence was not assessed using a validated scale and was instead interpreted indirectly through proxy indicators. Furthermore, the relatively short duration of the intervention limits conclusions regarding long-term sustainability. Despite these limitations, the findings provide valuable insights into the potential role of structured dietary interventions in weight management.

### Implications and future directions

The results highlight the potential of HDMS as a supportive strategy in obesity management, particularly when combined with dietary counseling. By addressing both practical and behavioral barriers to healthy eating, this approach may enhance adherence and improve clinical outcomes. Future research should focus on long-term effects, cost-effectiveness, and the scalability of such interventions across different populations and healthcare settings.

## Conclusion

This study demonstrates that HDMS effectively addresses barriers to dietary adherence, resulting in significant improvements in weight management, metabolic health, and eating behaviors compared to dietary counseling alone. The findings highlight the potential of structured, nutritionally balanced meal interventions to support individuals in achieving sustainable health outcomes. Further research is recommended to explore long-term benefits and broader applications of HDMS in obesity treatment.

## Data Availability

The data underlying this article will be shared on reasonable request to the corresponding author.
